# Heterologous Cytomegalovirus and Allo-Reactivity by Shared T Cell Receptor Repertoire in Kidney Transplantation

**DOI:** 10.3389/fimmu.2019.02549

**Published:** 2019-10-31

**Authors:** Lucia Stranavova, Ondrej Pelak, Michael Svaton, Petra Hruba, Eva Fronkova, Antonij Slavcev, Klara Osickova, Jana Maluskova, Petr Hubacek, Jiri Fronek, Petra Reinke, Hans-Dieter Volk, Tomas Kalina, Ondrej Viklicky

**Affiliations:** ^1^Transplant Laboratory, Institute for Clinical and Experimental Medicine, Prague, Czechia; ^2^CLIP – Childhood Leukaemia Investigation Prague, Department of Paediatric Haematology and Oncology, 2nd Faculty of Medicine, Charles University Prague and University Hospital Motol, Prague, Czechia; ^3^Department of Immunogenetics, Institute for Clinical and Experimental Medicine, Prague, Czechia; ^4^Department of Nephrology, Transplant Centre, Institute for Clinical and Experimental Medicine, Prague, Czechia; ^5^Department of Transplant Pathology, Transplant Centre, Institute for Clinical and Experimental Medicine, Prague, Czechia; ^6^Department of Paediatric Haematology and Oncology, 2nd Faculty of Medicine and Motol University Hospital, Charles University, Prague, Czechia; ^7^Department of Transplant Surgery, Institute for Clinical and Experimental Medicine, Prague, Czechia; ^8^BIH Centre for Regenerative Therapies, Berlin Centre for Advanced Therapies, Charité University Medicine Berlin, Berlin, Germany

**Keywords:** kidney transplantation, cytomegalovirus, ELISPOT, cross-reactivity, rejection, heterologous immunity, TCR repertoire

## Abstract

Cytomegalovirus (CMV) infection is associated with allograft rejection but the mechanisms behind are poorly defined yet. Although cross-reactivity of T cells to alloantigen and CMV has been hypothesized, direct evidence in patients is lacking. In this observational cohort study, we tested the pre-transplant effector/memory T cell response to CMV peptide pools and alloantigen in 78 living donor/recipient pairs using the interferon-gamma Enzyme-Linked ImmunoSpot (ELISPOT) assay. To prove the hypothesis of cross-reactivity, we analyzed by applying next-generation sequencing the T cell receptor ß (TCR- ß) repertoire of CMV- and alloantigen-reactive T cells enriched from peripheral pre-transplant blood of 11 CMV-seropositive and HLA class I mismatched patients. Moreover, the TCR-repertoire was also analyzed in the allograft biopsies of those patients. There was a significant association between the presence of pre-transplant CMV immediate-early protein 1 (IE-1)-specific effector/memory T cells and acute renal allograft rejection and function (*p* = 0.01). Most importantly, we revealed shared TCR-ß sequences between CMV-IE1 and donor alloantigen-reactive T cells in all pre-transplant peripheral blood samples analyzed in CMV-seropositive patients who received HLA class I mismatched grafts. Identical TCR sequences were also found in particular in post-transplant allograft biopsies of patients with concomitant CMV infection and rejection. Our data show the presence of functional, cross-reactive T cells and their clonotypes in peripheral blood and in kidney allograft tissue. It is therefore likely that CMV-donor cross-reactivity as well as CMV specific T cell elicited inflammation is involved in the processes that affect allograft outcomes.

## Introduction

Although the human cytomegalovirus (CMV) infection establishes a broad immunity which controls infection, rendering it asymptomatic in majority of immunocompetent hosts even if the CMV reactivates repeatedly following different stressors during life-time (1, 2). However, it might be associated with life-threatening complications in organ transplant recipients (3). Interestingly, it is widely acknowledged that, in addition to its direct pathogenic effects, CMV infection in organ transplant recipients is associated with more frequent acute and chronic rejection (4, 5). Although several possibilities have been proposed for those indirect negative effects, the mechanisms behind are poorly understood so far and direct proofs are missing. Persistent CMV infection elicits strong and lifelong T cell immunity that controls CMV reactivation/reinfection and prevents CMV disease by permanent dynamic interaction between the virus and the CMV-reactive T cell clones. However, CMV-reactive T cells can cause tissue damage by several mechanisms: (i) direct cytotoxic effect on CMV infected (allograft) cells, (ii) indirect bystander activation and proinflammatory milieu formation, and (iii) heterologous (cross-reactive) allorecognition (6).

The cross-reactivity of CMV-reactive effector T cells to HLA class I antigens has been discussed (7) and those cross-reactive cells were transiently found in the peripheral blood of kidney transplant recipients (8). T cell receptor (TCR) cross-reactivity has been suggested as primary means of increasing the effective size of T cell compartment, while cross-reactive memory cells have been shown to expand and activate more rapidly (9). Several mechanisms have been proposed for TCR cross-reactivity, including molecular mimicry and the ability of TCR to recognize different peptide-MHC complexes (10). Several other studies have shown the presence of cross-reactive virus-specific memory T cells and donor HLA molecules (7, 8, 11, 12). However, direct evidence of the role of heterologous TCR immunity in renal allograft rejection has not been shown so far.

Herein, we demonstrate that (i) the presence of CMV-reactive T cells pre-transplant predicts risk of acute allograft rejection, (ii) heterologous CMV- and donor-reactive cross-reactivity TCR-ß identical T cells pre-exists in patients prior to kidney transplantation, and (iii) identical cross-reactive T cell clones are detectable in renal allograft biopsies post-transplantation. Our data support the impact of heterologous immunity to CMV-IE1 and alloantigen among pre-transplant memory T cells on allograft outcome and indicate the need of adequate control not only by immunosuppression but also efficient antiviral strategies.

## Materials and Methods

### Patient Characteristics

In this observational cohort study, we evaluated the role of pre-transplant CMV-specific T cell immunity in acute rejection using an ELISPOT cohort consisting of 78 living donor kidney transplant recipients and their respective donors, all of whom underwent transplant surgery in the Institute for Clinical and Experimental Medicine in Prague between the years 2014 and 2017. The demographic data of the patients are summarized in Table 1. The peripheral blood of both donors and recipients was drawn pre-operatively to isolate PBMCs. All patients received tacrolimus, mycophenolate mofetil, and steroids as maintenance immunosuppression, initiated 48 h before the scheduled surgery. Patients at low immunological risk [panel-reactive antibody (PRA) <20%] received the anti-CD25 monoclonal antibody basiliximab (Simulect, Novartis), while patients at higher risk received rabbit polyclonal anti-thymocyte globulin (rATG, Thymoglobulin^®^, Genzyme Corporation) as induction immunosuppression. CMV prophylaxis with valganciclovir (Valcyte^®^, Roche) was given to CMV-seronegative recipients who had received grafts from seropositive donors or to CMV-seropositive recipients who had undergone rATG induction. Fourteen out of 78 patients experienced acute rejection episodes during the 1st year after transplantation and were treated as previously reported (13). For a detailed description of the histological findings, see Table S1.

**Table 1:** Demographics of the “ELISPOT” patient cohort.

	**Total**	**IE-1 positive**	**IE-1 negative**	***p***
Patients (*n*)	78	31	47	
Recipients age (years)^*^	45.6 ± 13.2	49.0 ± 11.7	43.0 ± 13.7	0.032
Donor age (years)^*^	48.6 ± 10.9	50.0 ± 11.1	48.0 ± 10.47	0.372
Gender of recipients (M/F)	54/24	20/11	34/13	0.322
Dialysis vintage (months)#	1.7 [0; 259.2]	4.0 [0; 259.2]	0.4 [0; 85.0]	0.424
HLA mismatch^*^	3.5 ± 1.41	3.7 ± 1.4	3.4 ± 1.4	0.427
PRA max (%)#	0 [0; 69]	0 [0; 69]	0 [0; 36]	0.932
PRA max ≥ 20% *n* (%)	12 (15.4)	6 (19.4)	6 (12.8)	0.430
Retransplantation *n* (%)	7 (8.9)	4 (12.9)	3 (6.4)	0.324
CMV prophylaxis *n* (%)	37 (47.4)	14 (45.2)	23 (48.9)	0.744
Pretransplant CMV IgG serostatus				
D+/R+	52 (66.7)	26 (83.9)	26 (55.3)	0.009
D+/R–	8 (10.3)	0 (0)	8 (17.0)	0.015
D–/R–	6 (7.7)	0 (0)	6 (12.8)	0.038
D–/R+	12 (15.4)	5 (16.1)	7 (14.9)	0.882
CMV DNAemia				
PCR > 10^2^ *n* (%)	9 (11.5)	6 (19.3)	3 (6.3)	0.079
Allo-positive ELISPOT *n* (%)	25 (32.1)	13 (41.9)	12 (25.5)	0.129
Induction Immunosuppression				
Basiliximab *n* (%)	49 (62.9)	19 (61.3)	30 (63.8)	0.151
Thymoglobulin *n* (%)	29 (37.1)	12 (38.7)	17 (36.2)	0.820
Rejection *n* (%)	14 (17.9)	11 (35.5)	3 (6.4)	0.001
eGFR 3M (mL/min)^*^	58.7 ± 12.6	53.2 ± 11.4	62.4 ± 12.2	0.003
eGFR 6M (mL/min)^*^	60.3 ± 13.7	55.0 ± 11.4	64.0 ± 13.9	0.006
eGFR 12M (mL/min)^*^	59.6 ± 13.5	55.5 ± 12.7	62.4 ± 13.5	0.119

*#Median [min; max]*.

* *Mean ± SD (range)*.

For the analysis of the TCR repertoire of CMV- and donor alloantigen-reactive T cells (the “cross-reactive” cohort) 11 donor/recipient pairs were selected with primary low risk and pre-transplant CMV-seropositive living donor renal allograft recipients from the years 2014 to 2016. For a summary of the demographic data of these patients, see Table 2. All patients received tacrolimus, mycophenolate mofetil, and steroids as maintenance immunosuppression (initiated 48 h before the scheduled surgery) and basiliximab as induction immunosuppression. All patients underwent a 3-month protocol kidney graft biopsy according to the centre's standard practice, while case biopsies were performed to histologically verify clinically suspected acute rejection. In 5 out of 11 patients, histological proven acute rejection episodes occurred within 3 months after the operation. Histological findings are given in Table S2. In the case of this patient cohort and their respective living donors, peripheral blood was drawn prior to transplantation in order to isolate PBMCs and the allograft biopsy was performed, with 2–3 mm of the tissue samples stored in Ambion RNAlater^®^ Stabilization Solution (Thermo Fisher Scientific) for future molecular evaluation.

**Table 2:** Demographics of the “cross-reactive” cohort.

**Patients (*n*)**	**11**
Recipients age (years)^*^	38.6 ± 13.7
Donor age (years)^*^	44.1 ± 13.4
Gender of recipients (M/F)	6/5
Dialysis vintage (months)#	3.9 [0; 25.9]
HLA mismatch^*^	3.7 ± 1.07
PRA max (%)#	0 [0; 13]
eGFR (mL/s)	1.25 ± 0.4
CMV prophylaxis *n* (%)	0 (0)
Pretransplant CMV IgG serostatus *n* (%)	
D+/R+	11 (100)
CMV DNAemia	
PCR > 10^2^ *n* (%)	3 (27.2)
Induction immunosuppression	
Basiliximab *n* (%)	11 (100)
Rejection *n* (%)	5 (45.5)
eGFR 3M (mL/min)^*^	67.9 ± 11.2
eGFR 6M (mL/min)^*^	75.0 ± 25.9
eGFR 12M (mL/min)^*^	74.6 ± 12.2

*#Median [min; max]*.

* *Mean ± SD (range)*.

All patients from the ELISPOT and cross-reactive cohorts as well as their respective donors gave their written informed consent to participate in the study. The local ethics committee approved the study protocol under No. G14-08-38.

### IFN-γ ELISPOT Assay

In the “ELISPOT” cohort, allo- and CMV-specific T cells were assessed using the IFN-γ ELISPOT method according to recently described protocols (14, 15). Peripheral blood mononuclear cells (PBMCs) were isolated from heparinized peripheral blood samples of donors and recipients taken prior to transplantation (using standard density gradient centrifugation) and cryopreserved in liquid nitrogen as described previously (16). After thawing, PBMCs were re-suspended with complete media [RPMI 1640 supplemented with 10% heat-inactivated fetal calf serum (FCS), penicillin + streptomycin (50 U/mL) and 1.7 mM sodium glutamate] and left for 24 h at 37°C in a CO_2_ incubator. Next, 3 × 10^5^ recipient PBMCs were stimulated with CD3-depleted donor cells to detect allospecific T cells, and with CMV antigens [whole protein-spanning overlapping peptide pools of immediate-early protein 1 (IE-1) and phosphoprotein 65 (pp65), length of each is 15 amino acids with 11 amino acid overlap] (Miltenyi Biotec) to detect CMV-reactive T cells [using pokeweed mitogen from Autoimmune Diagnostika (AID), GmbH as a positive control]. CMV peptide pools were used in the concentration of 1 μg/mL of pp65 or IE-1. PBMCs were seeded in an ELISPOT plate and incubated for 24 h at 37°C in a CO_2_ incubator. The ELISPOT kit used to detect IFN-γ-producing cells was obtained from AID. After 24 h incubation at 37°C in the CO_2_ incubator, cells were removed and the ELISPOT plate processed according to the manufacturer's protocol. The resulting numbers of spots were measured semi-automatically using an ELISPOT reader (AID iSpot FluoroSpot Reader System ELR07 IFL).

### Antigen Specific T Cells by Flow Cytometry and FACS Sorting

Antigen specific T cells (virus specific or donor reactive) were detected as proliferating CD8+ T cells by dye dilution technique. Eleven patients from the “cross-reactive” cohort were selected for flow cytometry sorting. Cryopreserved PBMCs from recipients and donors were thawed, suspended with complete media and left for 24 h at 37°C in a CO_2_ incubator. After resting, donor PBMCs were inactivated with Mitomycin C (50 μg/1 mL) (Sigma-Aldrich) for 2 h at 37°C in a CO_2_ incubator and washed twice with complete media (210 RCF/10 min). Afterwards, both recipient and donor PBMCs were labeled with the dilution dyes CellTrace™ Violet and Far Red Cell Proliferation kits (Thermo Fisher Scientific), respectively, according to the manufacturer's instructions. Labeled recipient PBMCs were aliquoted by 0.5 × 10^6^ in a 96-well plate (2 mL, V-bottom, Greiner Bio-One, GmbH) with a culture medium [RPMI 1640 supplemented with 10% heat-inactivated FCS, penicillin+ streptomycin (50 U/mL), 1.7 mM sodium glutamate, 0.00036% (v/v) β-mercaptoethanol, and 10 U/mL IL-2]. CellTrace™ Violet dilution dye labeled PBMC were stimulated with the following CMV antigens: 1 μg/mL of pp65, 1 μg/mL of IE-1 (Miltenyi Biotec), or whole CMV lysate (Vidia) for 6 days. To detect alloreactive and cross-reactive T cells, inactivated donor cells (ratio 1:1) were used as a stimulus (Far Red dye labeled). Additional controls consisting of unstimulated recipient cells and recipient cells with additional IL-2 (50 U/mL) (Sigma-Aldrich) were used to eliminate bystander cell proliferation (data not shown). After 6 days of stimulation, the cells were harvested in 5 ml tubes and washed once with PBS containing 2 mM EDTA. Antigen specific cells proliferate in response to antigen and loose their dilution dye (CellTrace Violet low cells in **Figure 2**). Washed cells were stained with CMV peptide-pentamers (Pro5^®^ MHC Pentamers, Proimmune) matched to the patient's HLA allele (Table S4) for 15 min at 4°C. Cells were washed once with PBS containing 2 mM EDTA and stained with antibodies against CD3 (anti-CD3 PC7, Beckman Coulter), CD4 (anti-CD4 ECD, Immunotech), CD8 (anti-CD8 APC-H7, BD Biosciences), and CD45 (anti-CD45 PerCP, Exbio) for 20 min at 4°C. As a subsequent step, cells were washed once with PBS and analyzed using the FACSAria™ III Cell sorter (BD Biosciences). Proliferating CellTrace™ Violet low cells were FACS sorted into a 50 μl elution buffer (Qiagen) (sort gate position is denoted as R1 gate in **Figure 2**). Sorted cells were then incubated in a thermo block heater at 99°C and at 500 rpm for 60 min to isolate DNA; the samples were subsequently stored at −20°C for next-generation sequencing (NGS) library preparation. FlowJo software (FlowJo, LLC) was used for flow cytometry data analysis.

To limit the possibility that cells stimulating the Allo responses contain CMV-infected cells, CMV seropositive donors' blood was examined for the presence of CMV genome by real-time quantitative PCR. DNA was extracted from whole blood using MagNA Pure Compact Nucleic Acid Isolation Kit (Roche, Basel, Switzerland), and the viral loads were normalized to 10,000 human genomic equivalents (17, 18). No CMV genome was found in those samples (Table S3).

### Kidney Biopsies and DNA Isolation

Kidney biopsy tissue samples taken from the 3-month protocol biopsies or from case biopsies due to deterioration of kidney graft function were available in 7 out of 11 patients from the “cross-reactive” patient cohort. All biopsy tissue samples were stored in Ambion RNAlater^®^ Stabilization Solution (Thermo Fisher Scientific) at −80°C. The tissue samples were thawed and genomic DNA isolated using the QIAamp DNA Micro Kit (Qiagen) according to the manufacturer's protocol. Isolated DNA were then stored at −20°C for NGS sequencing library preparation.

### TCR-β Repertoire Sequencing Using a Next Generation Sequencing Approach

Genomic DNA isolated from FACS-sorted cells and kidney biopsies were used for sequencing library preparation. Two-step PCR was used to detect the majority of V and J segments in complementarity-determining region 3 (CDR3) of the TCR-β sequence, as described previously (19–21). The established libraries were sequenced on the IonTorrent PGM using Hi-Q 400 bp chemistry (Thermo Fisher) and data were analyzed using the Vidjil application (VIDJIL web platform) (22). Samples with analyzed reads below 1,000 or reads in which the CDR3 sequence could not be identified were discarded from further analysis. Clones from the analyzed samples with reads lower than 10 were considered a source of possible cross-contamination between barcoded samples and were thus disregarded from the analysis. Non-productive rearrangements presumably originating from the second allele were retained in the analysis as additional markers of clonotypes.

### Statistical Analysis

Data were analyzed using GraphPad InStat 3 (GraphPad Software) and IBM SPSS 22 software. The normality of data distribution was tested using the Kolmogorov-Smirnov test. Since all data were shown not to correspond with standard normal distribution, only non-parametric statistical methods were used. The Mann-Whitney *U* and Chi-Square tests were used to compare the patient groups. Cox proportional hazards model were used to identify risk variables for rejection. The association of recipient, donor, and transplant parameters and immunological factors were first entered to univariate regression analyses (Table 3). All significant variables (*p* < 0.05) were included into final multivariate Cox model adjusted for induction treatment presenting the extent of immunological risk. Kaplan-Meier survival curves and the log-rank test were used to project rejection-free intervals and to compare groups. The Wilcoxon matched-pair signed-rank test was used to evaluate ELISPOT differences. To evaluate prediction of rejection risk on the basis of pretransplant pp65/IE-1/Allo ELISPOT receiver operating characteristic (ROC) curves and the calculation of the area under the curve (AUC) were used. Spearman's rank correlation coefficient was used to calculate correlations of pretransplant IE-1 ELISPOT and eGFR in 3, 6, and 12 months. All results with a *P*-value of <0.05 were considered statistically significant.

**Table 3:** Risk factors associated with graft rejection in univariate Cox regression.

**Univariate analysis variables**	**HR**	**95% CI**	***p***
Recipient age (years)	1.025	0.986–1.065	0.217
Recipient gender (male)	0.332	0.074–1.484	0.149
Donor age (years)	1.028	0.981–1.079	0.247
Donor gender (male)	7.320	0.960–55.96	0.055
Retransplantaion	1.162	0.362–3.733	0.801
HLA mismatch	0.984	0.680–1.425	0.933
PRA max	1.014	0.985–1.045	0.343
Dialysis vintage (months)	1.010	1–1.024	0.055
IE-1 ELISPOT	6.790	1.89–24.36	0.003
pp65 ELISPOT	1.001	0.999–1.004	0.300
Allo ELISPOT	0.986	0.963–1.010	0.255
CMV DNAemia PCR>10^2^	3.820	1.20–12.20	0.024
rATG induction treatment	2.360	0.66–8.48	0.187

*Hazard ratio (HR), 95% confidence interval (CI)*.

## Results

### Pre-transplant Presence of CMV-Specific T Cells Predicts Acute Rejection

**Figure 1:**
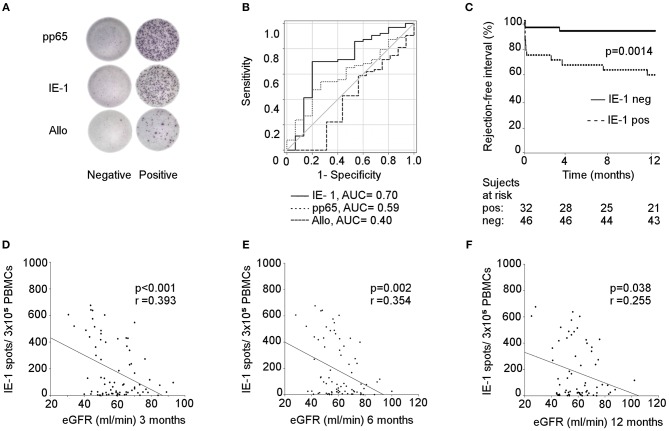
CMV-specific (but not allospecific) ELISPOT for predicting rejection and kidney allograft function. **(A)** visualization of IFN-γ spots after stimulation with pp65/IE-1/alloantigens in positive and negative recipients; **(B)** prediction of rejection risk based on the pre-transplant pp65/IE-1/allo ELISPOT; The operating characteristic (ROC) curves and the calculation of the area under the curve (AUC) were used for this analysis; IE-1: 95% confidence interval (CI): 0.54–0.87; *p* = 0.014; pp65: 95% CI: 0.44–0.74, *p* = 0.27; Allo: 95% CI: 0.27–0.59, *p* = 0.39; **(C)** rejection-free intervals of patients using IE-1-positive and -negative ELISPOTs expressed as Kaplan-Meier survival curves; *p* = 0.0014. Correlation between a pre-transplant IE-1 ELISPOT and eGFR at 3 **(D)**, 6 **(E)**, and 12 **(F)** months were established by Spearman's rank correlation coefficient; 3M: *p* < 0.001; 6M: *p* = 0.002; 12M: *p* = 0.038.

To evaluate the relation of pre-existing CMV-reactive and donor-alloreactive T cells and acute rejection of renal allografts, ELISPOT assays were performed in 78 consecutive patients. The design of the IFNγ-ELISPOT allows the detection only of memory/effector CD4+ and CD8+ T cells preformed *in vivo*. As expected, we observed significantly higher frequencies of pre-existing CMV-reactive than alloreactive memory/effector T cells (Figure 1A). Interestingly, the pre-transplant presence of a CMV-reactive response, both to IE-1 and pp65 whole protein overlapping peptide pools, had a stronger predictive power of acute rejection [IE-1 and pp65: AUC = 0.70, cut-off = 122.5 at 69.8% sensitivity and 80% specificity, 95% confidence interval (CI): 0.54–0.87; *p* = 0.014 and AUC = 0.59, cut-off = 332 at 63.5% sensitivity; and 53.3% specificity, 95% CI: 0.44–0.74, *p* = 0.27, respectively] than the donor-alloreactive ELISPOT (AUC = 0.40, cut-off = 25 at 66.7% sensitivity and 25.0% specificity, 95% CI: 0.27–0.59, *p* = 0.39, Figure 1B). Moreover, a shorter rejection-free interval was observed in patients with a positive pre-transplant IE-1 ELISPOT (Figure 1C).

Univariate Cox regression analysis revealed as significant risk factors of acute rejection only pretransplant positive IE-1 ELISPOT [Hazard ratio (HR) = 6.8, 95% CI: 1.89–24.36, *p* = 0.003] and post transplant positive CMV viral load by PCR>10^2^ (HR = 3.8, 95% CI: 1.2–12.2, *p* = 0.024) (Table 3). A multivariate Cox regression analysis adjusted for ATG induction treatment and CMV PCR > 10^2^ revealed only IE-1 positive ELISPOT (HR = 6.2, 95% CI: 1.67–22.3, *p* = 0.006) to be independent risk factor of acute rejection.

Interestingly, significant correlations were also found between pre-transplant IE-1 ELISPOTs and kidney graft function (estimated glomerular filtration rate (eGFR) using the Chronic Kidney Disease Epidemiology Collaboration (CKD-EPI) equation) at 3, 6, and 12 months (*p* < 0.001, *p* = 0.002, and *p* = 0.038, respectively) (Figures 1D–F). The demographic characteristics of patients with positive and negative IE-1 ELISPOTs are summarized in Table 1.

Taken together, CMV-reactive cellular immunity predicts acute rejection and short-term outcome of renal allografts.

### CMV- and Alloreactive T Cells Express Shared TCR Sequences

**Figure 2:**
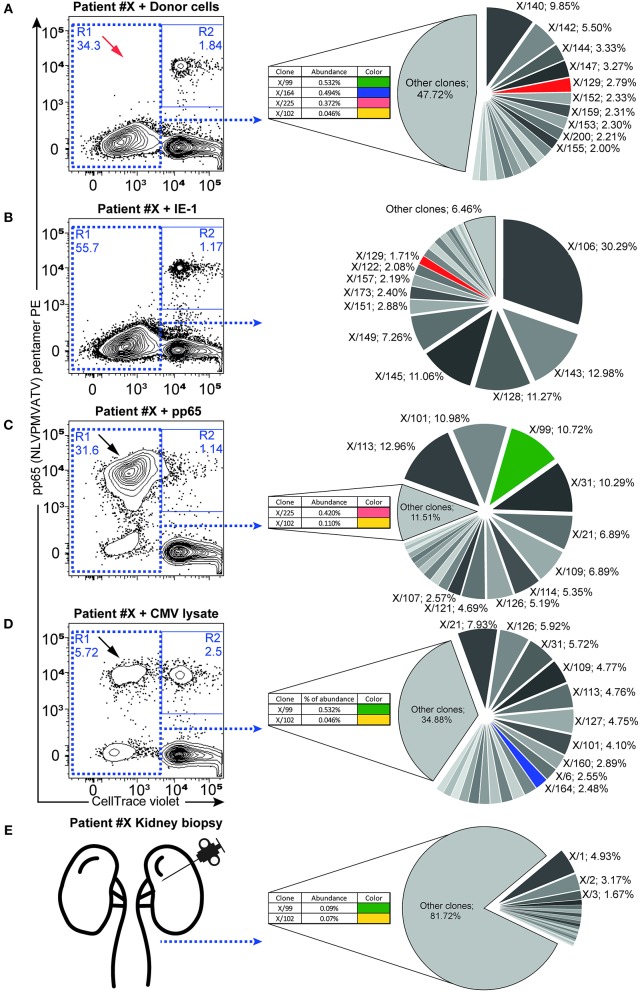
Antigen-responding CD8^+^ T cells containing cross-reactive TCR-β sequences. Antigen-specific responses were assessed as proliferating recipient CD8^+^ T cells (CellTrace low cells, plots show gated CD3+CD8+ lymphocytes) after 6-days of *ex-vivo* culture in combination with pentamer staining for the immunodominant (pp65: NLVPMVATV-specific) TCR receptor. Flow cytometry dot plots show the proliferation response of CD8^+^ T cells to donor cells **(A)**, IE-1 **(B)**, pp65 **(C)**, and whole CMV lysate **(D)**. Proliferating cells in R1 were FACS-sorted and used for subsequent NGS TCR-β repertoire analysis. Twenty of the most abundant TCR-β sequences are represented in the pie chart graph (right panels), while additional minor cross-reactive clones are shown in inlets. Color codes highlight the same TCR sequence clones found in the respective antigen-responding cells or in the kidney **(E)**. Black arrows highlight antigen-specific proliferating T cells recognizing the immunodominant pp65 peptide, with red arrows indicating their absence from the donor cell-elicited response. Relative clone abundance is shown next to the clone name in 10 of the most abundant clones or in the inlets. One representative patient (#X) is shown, with a complete list of responding cell fractions. The amount of sorted cells and available NGS reads are given in Table 4, while the cross-reactive clones found are listed in Table S5.

The strong association between the pre-transplant presence of CMV-reactive T cells and rejection prompted us to investigate the possible cross-reactivity of CMV-specific T cells to donor alloantigens by search for shared TCR sequences. First, we combined the donor alloantigen MLR with CMV-peptide pentamer staining to evaluate cross-reactivity at single cell level in pre-transplant peripheral blood mononuclear cells (PBMCs) (8). In contrast to previously published studies, we detected no T cells cross-reactive to the immunodominant pp65 CMV peptide and donor cells, as detected by the co-staining for CMV-pp65 pentamer and cell tracking dilution following proliferation to alloantigen stimulation (Figure 2A). However, the response to dominant epitopes (pentamer staining) is lower compared to the proliferative response to the whole CMV peptide pool as demonstrated in 10 out of 11 CMV-seropositive patients (Figures 2B–D and summarized in Figure S1). In parallel, donor alloreactive T cells were present in all patient samples (including CMV pentamer-negative ones) prior to transplantation.

To investigate whether cross-reactivity would be present among the total pool of CMV-reactive T cells, we isolated antigen-reactive T cells (either reactive to CMV peptide pool or donor PBMCs) by FACS sorting (Figures 2A–D) and performed NGS of TCR-β sequences (Table 4). In 10 out of 11 patients, we acquired a sufficient amount of reads for analysis. We hypothesize that while pentamer staining only reveals single immunodominant CD8+ T cell clones, cross-reactivity may be caused by other less-dominant TCR clones. We were able to identify hundreds of distinct TCR-ß sequences from sorted antigen-reactive T cells from all patients (median 392 [241; 491], Table S5). Indeed, multiple clones sharing the same unique TCR-ß sequences were found in both CMV- and donor-reactive samples (Figure 2, right panels) from all patients, regardless of occurrence of rejection (Table S6).

**Table 4:** Percentages of proliferating CD8^+^ T cells (% CellTrace low), the number of sorted proliferating CD8^+^ T cells (sorted events), and the number of reads obtained after TCR-β next generation sequencing of sorted proliferating CD8^+^ T cells (No. of reads) in response to different stimulations.

**Stimulation**	**Donor PBMCs cells**			**IE-1**			**pp65**			**CMV lysate**			**Kidney**
**Patient ID**	**% CellTrace low**	**Sorted events**	**No. of reads**	**% CellTrace low**	**Sorted events**	**No. of reads**	**% CellTrace low**	**Sorted events**	**No. of reads**	**% CellTrace low**	**Sorted events**	**No. of reads**	**No. of reads**
I	35.5	11,142	33,778	49.6	13,014	28,014	31.2	5,258	13,246	12.3	1308	53188	16423
II	8.93	1,888	28,855	63.2	20,656	802	65.2	29,571	19,164	49.1	14738	3846	NA
III	36.1	10,516	10,804	36	3,081	56,507	16.9	933	71,615	15.6	1267	65789	12312
IV	33.6	12,458	35,175	52.6	7,945	51,790	41.5	8,673	25,332	7.5	2263	61281	NA
V	10.4	2,788	27,888	3.2	218	20,024	46.3	6,339	31,116	37.3	7534	16768	6915
VI	13.7	3,262	68,617	10.6	2,000	65,951	40.3	3,546	70,503	12.9	3194	104427	121
VII	24.3	3,428	46,562	57.4	70,911	1,702	10.1	2,901	51,597	8.3	5410	79025	20816
VIII	24.7	23,849	1,549	38.7	22,871	9,790	41.3	19,032	4,141	16.4	931	53528	20825
IX	36.5	3,731	37,273	0.1	8	0	7.75	302	33,931	2.8	200	56879	2799
X	34.3	18,084	26,346	55.7	20,473	1,898	31.6	11,140	32,570	5.7	2040	66330	19806
XI	7.7	2,289	70,817	67.9	18,835	140	NA	NA	NA	67.3	37150	143	NA

*The kidney column only lists results from TCR-β next generation sequencing of isolated cells from fine needle biopsies where sorting of CD8^+^ T cells was not possible*.

Our results also provide evidence that both donor cells and CMV antigens can trigger identical T cell clones for proliferation showing functional responsiveness.

### Shared Cross-Reactive TCR-ß Clonotypes Are Detectable in Renal Allograft Biopsies

Next, we sought to investigate whether cross-reactive TCR-ß clonotypes would be detectable in the allografts. Allograft biopsy samples were made available for 7 patients investigated for alloreactive and CMV-specific clonotypes (see “cross-reactive” cohort described above). In the kidney biopsy samples of 6 out of 7 patients, we were able to find identical TCR-β CDR3 sequences as in the alloreactive T lymphocytes pre-transplant. For the remaining patient, only 16 clones could be analyzed from the sequencing results of the MLR tube. Therefore, cross-reactive clones could have been missed due to the lower coverage of this sequencing library. In parallel, CMV-reactive TCR-ß clonotypes were found in the biopsy samples even at higher frequencies in the same patients, with a median of 3 clones per patient and a maximum of 11 (Table 5). The CMV-reactive clonotypes in the kidney covered 0.5–6.4% of all TCR-β sequences (Table 5) found in the kidney biopsies (see individual clones in Table S6). Finally, in 3 out of 7 biopsy samples, we detected CMV/donor alloantigen cross-reactive clonotypes identified pre-transplant in peripheral blood (Table 5).

**Table 5:** CMV-, Allo-, and Cross-reactive clones identified from blood pre-transplant are found in the kidney.

**Patient ID**	**Number of shared clones between PBMCs and kidney from kidney**		
	**CMV specific (% of reads from all TCR-β sequences found in the biopsy)**	**Alloreactive (% of reads from all TCR-β sequences found in the biopsy)**	**Cross-reactive (% of reads from all TCR-β sequences found in the biopsy)**
I	1 (0.5%)	1 (0.4%)	0%
III	1 (0.7%)	3 (2.4%)	2 (1.8%)
V	3 (1.8%)	6 (12.6%)	2 (3%)
VII	3 (0.5%)	2 (0.6%)	0%
VIII	11 (5.1%)	0%	0%
IX	0%	2 (9.2%)	0%
X	1 (0.1%)	7 (6.4%)	1 (0.1%)

*The percentage of reads from clones that were identified in the functional assay as CMV-reactive, Allo-reactive, or in both tubes as Cross-reactive clones is shown as fraction of all the TCRβ sequences found in the kidney biopsies*.

Remarkably, in agreement with the acknowledged capacity of CMV-reactive T cell clones to expand upon CMV reactivation, cumulative abundance of CMV-reactive TCR-ß sequences was the highest (6.4 and 5.1%) in the two kidney tissue samples obtained from patients (No. VIII and X) suffering from significant CMV reactivation (viral load: 935 copies/ml and 1,020 copies/ml of plasma, respectively) concomitantly with biopsy-proven cellular rejection.

In summary, CMV-reactive and alloreactive clonotypes were found in all allograft biopsies patients analyzed, and in 3 out of 7 patients, we detected cross-reactive clonotypes as defined by pre-transplant analyses. Importantly, the highest number of these cross-reactive clones was observed in the two patients with CMV reactivation and concomitant cellular rejection.

## Discussion

The immunity in response to previous virus infections can modify the immune response to other antigens. Although heterologous immunity can be beneficial by boosting protective responses, it can also result in severe immunopathologies (6). Here, for the first time, we provide evidence that heterologous immunity can be detected in blood and biopsies of renal allograft recipients. Firstly, we found that high frequencies of CMV-reactive effector/memory (but not of alloreactive) T cells detected pre-transplant were associated with subsequent occurrence of T cell-mediated rejection. Secondly, multiple cross-reactive T cell clones (shared TCR-ß sequences) were found in both CMV- and donor-reactive T cells enriched from pre-transplant peripheral blood samples. Finally, TCR-ß sequences of alloreactive [CMV-reactive, and cross-reactive (CMV & MLR)] clonotypes were found in renal allografts; the latter particularly in association with CMV-associated T cell mediated acute rejection. Therefore, our data demonstrate that identical clonotypes of T cells can react in response to alloantigens as well as CMV antigens. This observation might explain how CMV reactivation, especially in the case of high viral load during uncontrolled replication, boosts directly not only the CMV but also the alloimmune T cell response.

Interestingly, in contrast to the association between high levels of pre-transplant CMV memory/effector response, in our study, we found no associations between pre-transplant donor-reactive memory/effector T cell response and acute rejection. Given the standardized, validated and robust ELISPOT method applied (previously used in a large European multicentre clinical trial; see www.biodrim.eu), it is unlikely that any methodological bias occurred. An earlier study reported that higher pre-transplant T cell alloresponse was associated with acute allograft rejection in a study where patients received non-lymphocyte-depleting induction immunosuppression (23). Contrary, in our study some 40% of patients had received rATG T cell depletive induction immunosuppression. Similarly, pre-transplant allo-T cell responses have also been shown to correlate with lower post-transplant eGFR in patients with non-depleting induction (24). Apart from the association between CMV-specific memory/effector T cells and acute rejection, we found significant correlation with lower post-transplant eGFR at three different time-points during the first post-Tx year, rendering our observations more robust. Therefore, it is likely that the T cell-depletion strategy used in about half of our patients effectively reduced the available clonal size of alloreactive memory/effector T cells to a level that could be further controlled by maintenance immunosuppression.

Interestingly, there was weaker association of CMV-pp65- vs. CMV-IE-1-reactive T cells with acute rejection in our study. This phenomenon might be explained by higher CD8+ T cell response to IE-1 than to pp65 antigens (25).

In fact, subclinical CMV reactivation is frequently detected in over 30% of kidney transplant recipients despite CMV prophylaxis (26–28). We speculate that subtle localized CMV reactivations are even more frequent and, while undetected, provide antigen stimulation to CMV-specific T cells. This is in line with observations made by authors of a previous prospective randomized trial. They found that late-onset of CMV viremia, which developed in more than half of patients despite CMV prophylaxis, is associated with poorer outcomes (29).

Although it was reported that at least 151 of the 213 predicted CMV proteins, elicited T cell responses in at least one out of 33 donors (30), we and others could show that the T cell responses to IE-1 and pp65 CMV-proteins are the most dominant ones. Therefore, we concentrated in this study on the two immunodominant CMV proteins.

Applying the previously described method for detecting cross-reactive T cells based on MLR-reactivity combined with peptide-pentamer staining, was not effective in our scenario to detect cross-reactive T cells (7, 8, 31). One reason for this might be the use of unbiased PBMC samples with a scarcity of cross-reactive cells. In fact, as our access to patient material was limited by ethical reasons, we used only 5 × 10^5^ T lymphocytes for functional stimulation, resulting after sort in limited yield of antigen-reactive T cells ranging from 3,846 to 104,427 and 1,888 to 23,849 CMV- and allospecific-proliferating T cells, respectively, for TCR repertoire analysis. Moreover, our recent data show that immunodominant epitopes for one particular HLA-type, as detected and enriched by peptide/dextramer staining, do not reflect the whole response to a particular CMV protein. Therefore, we developed recently the method of T cell stimulation by whole protein-spanning overlapping peptide pools covering almost all epitopes in a HLA-independent matter (32). Applying this method here, we could detect all three categories of CMV-, donor alloantigen-, and cross-reactive T cells in all patients with sufficient yield after sorting derived from pre-transplant blood samples despite limited amounts of reactive T cells (and resulting reads in NGS). These results show the potency of recipients' memory/effector T cell pool to react in case of CMV reactivation post-transplantation with both a protective CMV-specific and a putatively harmful CMV/allo-cross reactive response. In other words, CMV reactivation because of breakthrough through or after weaning of antiviral prophylaxis that might be amplified by TNF-release following ATG application can trigger putatively harmful alloresponse by crossreactivity (33). In line with this, we could detect cross-reactive TCR-ß clonotypes in the kidney biopsies of 3 out of 6 patients with sufficient yield for analysis. Whether the absence of detectable shared cross-reactive TCR-ß sequences in the remaining three biopsy samples is due to sensitivity problems or missing triggering by CMV is not clear, but the high abundancy in the samples just of the two patients suffering from enhanced CMV viral load and concomitant acute rejection supports their pathogenic role in CMV-associated graft injury.

In summary, our data show that within the large peripheral population of CMV-specific memory T cells there is a pool of cross-reactive T cell clonotypes that can produce effector T cells capable of migrating into kidney allografts. Moreover, these T cell clonotypes (when in the presence of chronic antigenic stimuli, such as CMV) may be susceptible to enhanced proliferation and allograft rejection. This phenomenon seems to be universal and corresponds with previous hypotheses about the cross-reactive virus-alloimmune response (12, 34). Specific allo-HLA cross-reactivity has been reported for EBV, CMV, varicella-zoster virus (VZV), and influenza A virus-specific T cells at clonal level, while cross-reactivity has been shown to be mediated by the same TCRs (35, 36). However, our data demonstrate for the first time their occurrence in the unbiased bulk T cell pool from peripheral blood and intragraft.

The limitations of this study must also be acknowledged. The analysis was confined by the limited number of patients and TCR-β chains; furthermore, TCR-α rearrangements were not examined. The configuration of TCR-β chains (including D segments) building in particular the CDR3 region ensures much greater variability of rearranged sequences than TCR-α. Therefore, TCR-β is considered more informative than TCR-α and has been widely used in similar studies. Aside of T cells several other cells (e.g., NK cells) may produce IFNγ after stimulation. Therefore, we phenotypized IFNγ-producing cells stimulated by CMV antigens by flow cytometry. However, the majority of IFNγ-producing cells were T lymphocytes (55%), while NK cells accounted for 5% of IFNγ-producing cells only. The “cross-reactive cohort” subjected TCR- β NGS comprised only by CMV seropositive donor-recipient pairs. Among CMV seropositive-donors cells the CMV-infected cells might be present (37). To minimize the risk of potential activation by CMV infected donor cells, we evaluated those donors for the presence of CMV in their peripheral blood and found none CMV genome.

Our data show that CMV-specific cellular response pre-transplant predicts rejection and document that surprisingly large proportion of patients harbors CMV and donor cross-reactive clones. CMV and donor cross-reactive T cells might thus directly damage the donor cells, being expanded by CMV antigenic stimulation during CMV reactivation. This effect might be supported by CMV specific response that builds inflammatory environment in the kidney. We recommend the approaches aimed at preventing CMV reactivation to be employed more aggressively; not only to prevent CMV disease but also to limit cross-reactivity-induced graft rejection.

In conclusion, we report that in our patient cohort the presence of cytomegalovirus IE-1-specific memory/effector IFN-gamma secreting T cells predict kidney transplant rejection and poorer 1 year graft function. Since we established the presence of functional, cross-reactive T cells and their clonotypes in peripheral blood, tracking the clonotypes directly in the kidney tissue, it is therefore likely that CMV-donor cross-reactivity as well as CMV specific T cell elicited inflammation is involved in the processes that affect allograft outcomes. Future studies should be carried out to determine whether more aggressive prevention and treatment of CMV reactivation might possibly limit alloimmune injury boosted by cross-reactive T cells.

## Data Availability Statement

All datasets generated for this study are included in the article/Supplementary Material.

## Ethics Statement

The study protocol was approved by the Ethics Commintee of the Institute for Clinical and Experimental Medicine and Thomayer Hospital under number: G14-08-38. All subjects gave written informed consent in accordance with the Declaration of Helsinki.

## Author Contributions

OV and TK share senior authorship designed and supervised the research and wrote the manuscript. LS, OP, MS, PHr, AS, and EF performed the research, participated in the data analysis, and manuscript writing. JF, JM, PHu, and KO helped with carrying out the research. PR and H-DV helped in establishment of the ELISPOT technology and significantly contributed to writing the manuscript.

### Conflict of Interest

The authors declare that the research was conducted in the absence of any commercial or financial relationships that could be construed as a potential conflict of interest.

## Abbreviations

TCR: T cell receptor
ELISPOT: Interferon-gamma Enzyme-Linked ImmunoSpot
IE-1: CMV immediate-early protein 1
pp65: Phosphoprotein 65
CI: Confidence interval
eGFR: Estimated glomerular filtration rate
CKD-EPI: Chronic Kidney Disease Epidemiology Collaboration equation
MLR: Mixed lymphocyte reaction
PBMCs: Peripheral blood mononuclear cells
NGS: Next-generation sequencing
VZV: Varicella-zoster virus
rATG: Rabbit polyclonal antithymocyte globulin
PRA: Panel-reactive antibody
CDR3: Complementarity-determining region 3
HR: Hazard ratio
FCS: Fetal calf serum
ROC: Receiver operating characteristic curve
AUC: Area under the curve.
