# Supplementary material for: Heterologous Cytomegalovirus and Allo-Reactivity by Shared T Cell Receptor Repertoire in Kidney Transplantation
Source: Front Immunol. 2019 Oct 31;10:2549. doi: 10.3389/fimmu.2019.02549 (PMC6834532; doi:10.3389/fimmu.2019.02549)
Supplement: Supplementary file 1 [file Data_Sheet_1.docx]

Supplementary Material

| Supplementary Figures |
| --- |
| Figure S01: Overview of all patients showing their responses to different stimulations. |
| Supplementary Tables |
| Table S01: Banff classification of rejection biopsies categorized as sets of the “ELISPOT” cohort |
| Table S02: Banff classification in biopsies with rejection within sets of the “Cross-reactive” cohorts of patients  Table S03: Number of CMV viral load measured in the whole blood from CMV seropositive donors |
| Table S04: Pentamers used for detecting CMV-specific T cells |
| Table S05: Number of clones analyzed from particular specimens. Number of cross-reactive clones identified in both MLR and CMV induced specimens is shown together with the percentage of reads belonging to these clones out of all TCRβ sequences in the MLR tube |
| Table S06: Table of all common clones (found among CMV specific cells and in the Kidney). Patient ID is given in roman numerals. Clone ID is given as Arabic numeral |

**Supplementary Figures**

**Figure S01:** Overview of all patients showing their responses to different stimulations.
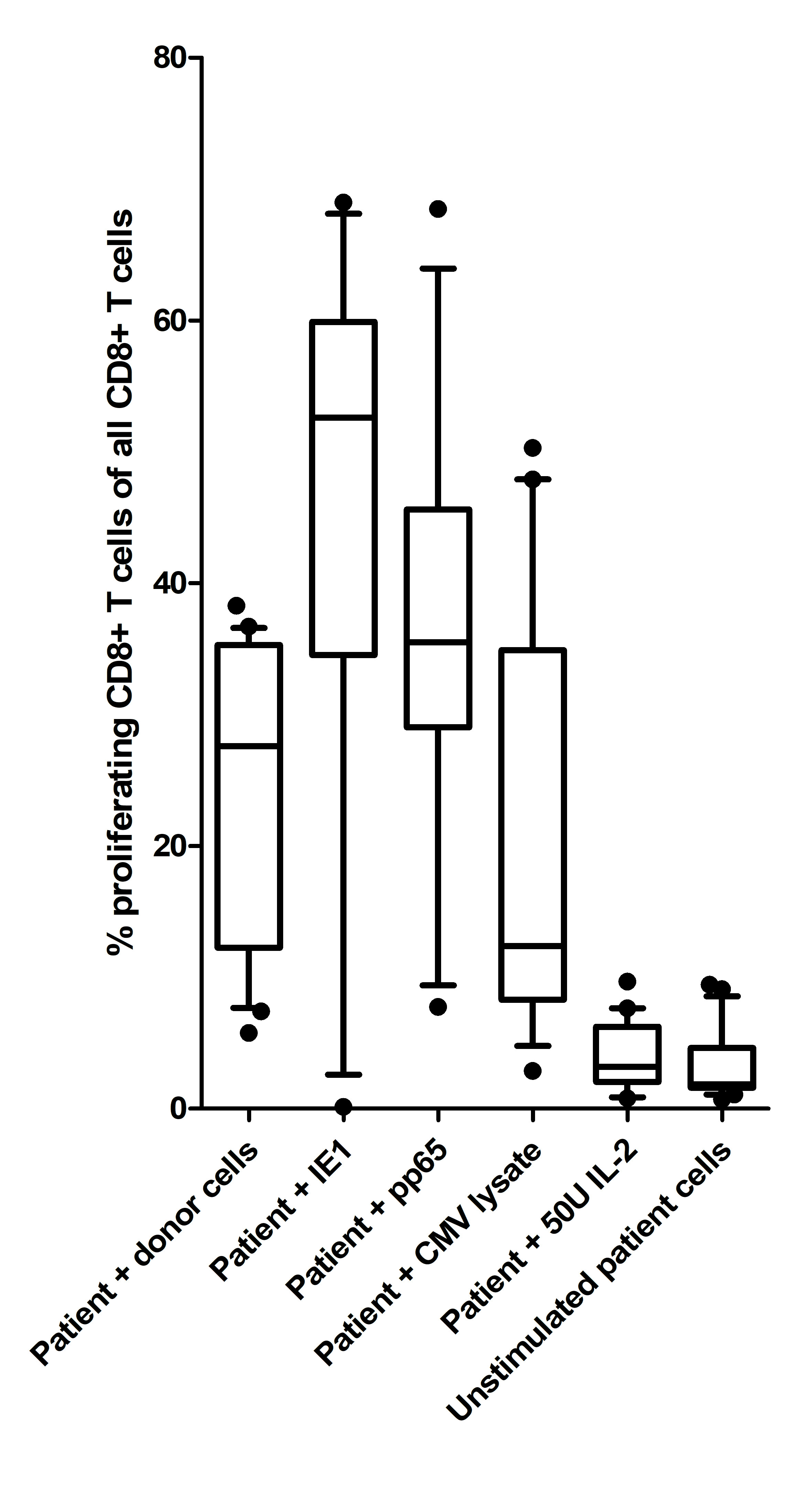


**Supplementary Tables**

**Table S01**: Banff classification of rejection biopsies categorised as sets of the “ELISPOT” cohort

| “ELISPOT” cohort | | | | | | | | | | | | | | | | | |
| --- | --- | --- | --- | --- | --- | --- | --- | --- | --- | --- | --- | --- | --- | --- | --- | --- | --- |
| Pat. | **Diagnosis** | **g** | **cg** | **mm** | **i** | **t** | **ti** | **ci** | **ct** | **v** | **cv** | **ah** | **aah** | **ptc-s** | **ptc-q** | **ptc-e** | **C4d** |
| I | TCMR 1A | 0 | 0 | 0 | 2 | 2 | 2 | 0 | 1 | 0 | 0 | 0 | 0 | 0 | 0 | 0 | 0 |
| II | TCMR IA | 0 | 0 | 0 | 2 | 2 | 2 | 0 | 1 | 0 | 1 | 1 | x | x | x | x | x |
| III | TCMR IB | 0 | 0 | 0 | 2 | 3 | 1 | 1 | 1 | 0 | 0 | 1 | x | x | x | x | 0 |
| IV | TCMR IIA | 0 | 0 | 0 | 0 | 1 | 0 | 0 | 1 | 1 | 2 | 1 | 0 | 0 | 0 | 0 | 0 |
| V | TCMR IA | 1 | 0 | 0 | 1.5 | 2 | 2 | 1 | 1 | 0 | 1 | 2.5 | 2 | 0 | 0 | 0 | 0 |
| VI | AMR | 0 | 0 | 0 | 0 | 0 | 0 | 1 | 1 | 0 | 0 | 1.5 | x | x | x | x | 2 |
| VII | TCMR IIA | 2 | 0 | 0 | 2 | 3 | 2 | 1 | 1 | 1 | 1 | 1 | 1 | 0 | 0 | 0 | 0 |
| VIII | TCMR IIA | 0 | 0 | 0 | 2 | 3 | 2 | 1 | 1 | 0 | 1 | 1 | 0 | 0 | 0 | 0 | 0 |
| IX | TCMR IIB | 1 | 1 | 1 | 0 | 0 | 2 | 1 | 1 | 2 | 1 | 2 | 1 | 1 | 1 | 1 | 3 |
| X | TCMR IB | 0 | 0 | 0 | 2 | 3 | 2 | 1 | 1 | 0 | 1 | 1 | 0 | 2 | 2 | 1 | 0 |
| XI | TCMR IB | 0 | 0 | 0 | 2 | 3 | 2 | 1 | 1 | 0 | 1 | 1 | 0 | 0 | 0 | 0 | 0 |
| XII | TCMR IB | 0 | 0 | 0 | 2 | 3 | 2 | 1 | 0 | 0 | 1 | 1 | 1 | 0 | 0 | 0 | 0 |
| XIII | AMR | 1 | 0 | 0 | 0 | 0 | 0 | 1 | 1 | 0 | 1 | 1 | 0 | 0 | 0 | 0 | 3 |
| XIV | TCMR IB | 1 | 0 | 0 | 2 | 3 | 2 | 1 | 1 | 0 | 2 | 2 | 1 | 0 | 0 | 0 | 1 |

Abbreviations according Banff classification[^1^](#_ENREF_1): T cell-mediated rejection (TCMR), Antibody-mediated rejection (AMR), Glomerulitis score (g), Chronic glomerulopathy score (cg), Mesangial matrix expansion (mm), Inflammation (i), Tubulitis (t), Total inflammation (ti), Interstitial fibrosis (ci), Tubular atrophy (ct), Intimal arteritis (v), Vascular fibrous intimal thickening (cv), Arteriorlar hyalinosis (ah), Hyaline arteriolar thickening (aah), Peritubular capillaritis score (ptc), Complement component 4d (C4d)

**Table S02**: Banff classification in biopsies with rejection within sets of the “Cross-reactive” cohorts of patients

| “Cross-reactive” cohort | | | | | | | | | | | | | | | | | |
| --- | --- | --- | --- | --- | --- | --- | --- | --- | --- | --- | --- | --- | --- | --- | --- | --- | --- |
| Patient | **Diagnosis** | **g** | **cg** | **mm** | **i** | **t** | **ti** | **ci** | **ct** | **v** | **cv** | **ah** | **aah** | **ptc-s** | **ptc-q** | **ptc-e** | **C4d** |
| VII | TCMR IIA | 0 | 0 | 0 | 1 | 1.5 | 2 | 1 | 1 | 1 | 1 | 1 | 0 | 0 | 0 | 0 | 0 |
| VIII | TCMR IB | 0 | 0 | 0 | 2 | 3 | 2 | 1 | 1 | 0 | 1 | 1 | 0 | 0 | 0 | 0 | 0 |
| IX | TCMR IIA | 2.5 | 0 | 0 | 0.5 | 3 | 2 | 1 | 1 | 1 | 1.5 | 0 | 0 | 2 | 2 | 2 | 1 |
| X | TCMR IB | 0 | 0 | 0 | 3 | 3 | 3 | 0.5 | 1 | 0 | 2 | 1 | 0 | 0 | 0 | 0 | 1 |
| XI | TCMR IB | 1 | 0 | 0 | 2 | 3 | 2 | 0 | 1 | 0 | 2 | 1 | 0 | 2 | 2 | 2 | 3 |

Abbreviations according Banff classification[^1^](#_ENREF_1): T cell-mediated rejection (TCMR), Antibody-mediated rejection (AMR), Glomerulitis score (g), Chronic glomerulopathy score (cg), Mesangial matrix expansion (mm), Inflammation (i), Tubulitis (t), Total inflammation (ti), Interstitial fibrosis (ci), Tubular atrophy (ct), Intimal arteritis (v), Vascular fibrous intimal thickening (cv), Arteriorlar hyalinosis (ah), Hyaline arteriolar thickening (aah), Peritubular capillaritis score (ptc), Complement component 4d (C4d)

**Table S03:** CMV viral loads measured in the whole blood of CMV-seropositive donors used in cross-reactivity experiment

| Donors Nr. | Genome equivalent/uL DNA | CMV* |
| --- | --- | --- |
| #I | 16746 | 0 |
| #II | 10395 | 0 |
| #III | 20011 | 0 |
| #IV | 12185 | 0 |
| #V | 11294 | 0 |
| #VI | 12990 | 0 |
| #VII | 10785 | 0 |
| #VIII | 9362 | 0 |
| #IX | 16604 | 0 |
| #X | 11367 | 0 |
| #XI | NA | NA |

*Viral load threshold listed in exponential form, normalized on 10 000 human genome equivalent.

**Table S04**: Pentamers used for detecting CMV-specific T cells

| Patient | HLA –A; -B (R) | HLA –A; -B (D) | Pentamers used for detecting CMV specific cells | % pentamer positive CD8^+^ cells |
| --- | --- | --- | --- | --- |
| I | A1,3; B7,8 | A3,24; B7,61 | A*01-pp65; B*07-pp65; B*08-IE-1 | 1.07; 0.58; 2.68 |
| II | A2,11; B7,62 | A3,25; B7,18 | A*02-IE-1; A*02-pp65 | 0.35; 0.36 |
| III | A2,24; B18,27 | A2,3; B7,44 | A*02-IE-1; A*02-pp65 | 0; 1.09 |
| IV | A2,30; B13,18 | A1,26; B62,35 | A*02-IE-1; A*02-pp65 | 0.14; 0.37 |
| V | A1,2; B80,60 | A2,66; B62,60 | A*01-pp65; A*02-IE-1; A*02-pp65 | 0.06; 0.21; 1.18 |
| VI | A3,24; B60,51 | A1,24; B44,51 | A*03-IE-1 | 0.04 |
| VII | A2,3; B18,38 | A1; B8,13 | A*02-IE-1; A*02-pp65 | 0.01; 0.12 |
| VIII | A1,68; B7,60 | A2,68; B18,60 | A*01-pp65; B*07-pp65 | 0; 0.33 |
| IX | A11,25; B8,35 | A2,26; B62,50 | B*08-IE-1 | 0.07 |
| X | A2; B18,27 | A26,32; B8,56 | A*02-IE-1; A*02-pp65 | 0.03; 3.13 |
| XI | A2,32; B7,44 | A3,29; B7,44 | A*02-IE-1; A*02-pp65 | 0.02; 0.26 |

**Table S05:** Number of clones analysed from particular specimens. Number of cross-reactive clones identified in both MLR and CMV induced specimens is shown together with the percentage of reads belonging to these clones out of all TCRB sequences in the MLR tube.

| Patient ID | Total | MLR | IE1 | p65 | CMV | Kidney | Cross reactive MLR&CMV | Cross reactive MLR&CMV (% of MLR) |
| --- | --- | --- | --- | --- | --- | --- | --- | --- |
| I | 491 | 98 | 84 | 84 | 92 | 97 | 15 | 43.35% |
| II | 241 | 82 | NA | 13 | 18 | NA | 1 | 0.08% |
| III | 478 | 100 | 52 | 114 | 95 | 99 | 3 | 4.71% |
| IV | 320 | 102 | 78 | 43 | 91 | NA | 31 | 43.43% |
| V | 344 | 98 | 33 | 29 | 35 | 92 | 3 | 1.14% |
| VI | 477 | 101 | 90 | 83 | 91 | NA | 4 | 0.96% |
| VII | 437 | 94 | 2 | 69 | 88 | 101 | 2 | 1.23% |
| VIII | 431 | 16 | 44 | 37 | 92 | 105 | 1 | 0.97% |
| IX | 328 | 101 | NA | 42 | 27 | 48 | 5 | 4.22% |
| X | 353 | 85 | 18 | 42 | 62 | 102 | 3 | 1.07% |
| XI | NA | NA | NA | NA | NA | NA | NA | NA |

**Table S06:** Table of all common clones (found among CMV specific cells and in the Kidney). Patient ID is given in roman numerals. Clone ID is given as Arabic numeral.

| Patient/ Clone ID | Identified clone | CDR3 (AA) | % of all donor reactive TCRβ clones in peripheral blood | % of all CMV reactive TCRβ clones in peripheral blood | % of all TCRβ clones in kidney post transplant |
| --- | --- | --- | --- | --- | --- |
| A. CMV clones found in kidney | | | | | |
| I/5 | TRBV4-2*01 1/TGGGAGAGGGA/5 TRBJ1-1*01 | CASSQVGEGTEAFF | 0% | 1.17% | 0.47% |
| III/169 | TRBD1*01 5/CTAG/2 TRBJ2-7*01 | n.d. | 0% | 0.57% | 0.66% |
| V/1 | TRBV3-1*01 0/AGGGACAG/2 TRBJ1-6*01 | CASSQEGTAYNSPLHF | 0% | 42.41% | 0.51% |
| V/7 | TRBV29-1*01 3/GGGAGGACAGTT/3 TRBJ2-7*01 | CSVEGGQFYEQYF | 0% | 13.48% | 0.32% |
| V/108 | TRBV28*01 3/GGGTTTACA/0 TRBJ1-3*01 | CASSLGLHSGNTIYF | 0% | 0.15% | 0.92% |
| VII/17 | TRBV20-1*02 1/CAAAACGAAGACTAGCGGGAATCCTTAT/2 TRBJ2-5*01 | CSAKTKTSGNPYQETQYF | 0% | 2.51% | 0.38% |
| VII/395 | TRBV2*01 1/ATTCAGGG/4 TRBJ2-7*01 | CASSEDSGYEQYF | 0% | 0.29% | 0.06% |
| VII/422 | TRBD1*01 2/T/4 TRBJ2-1*01 | n.d. | 0% | 0.26% | 0.06% |
| VIII/6 | TRBV4-3*01 4/CGAGTCGA/2 TRBJ1-1*01 | CASSPSRNTEAFF | 0% | 2.89% | 1.88% |
| VIII/8 | TRBD2*01 1//5 TRBJ2-1*01 | n.d. | 0% | 9.87% | 0.26% |
| VIII/9 | TRBV4-3*01 4/CCGCCCGC/2 TRBJ1-1*01 | CASSPARNTEAFF | 0% | 0.31% | 1.49% |
| VIII/15 | TRBV27*01 3/GGGGCCAG/3 TRBJ1-6*02 | CASSLGPANNSPLHF | 0% | 6.13% | 0.06% |
| VIII/20 | TRBD1*01 1//2 TRBJ1-4*01 | n.d. | 0% | 5.97% | 0.09% |
| VIII/41 | TRBD2*02 10/GGT/0 TRBJ2-7*01 | n.d. | 0% | 0.60% | 0.47% |
| VIII/54 | TRBV5-5*01 28//36 TRBJ1-5*01 | n.d. | 0% | 0.04% | 0.11% |
| VIII/72 | TRBD1*01 5/CGA/1 TRBJ1-1*01 | n.d. | 0% | 0.53% | 0.16% |
| VIII/83 | TRBD2*02 6//2 TRBJ2-1*01 | n.d. | 0% | 1.17% | 0.29% |
| VIII/91 | TRBD1*01 0/GCGC/7 TRBJ1-2*01 | n.d. | 0% | 0.75% | 0.15% |
| VIII/100 | TRBD1*01 1//3 TRBJ1-1*01 | n.d. | 0% | 0.16% | 0.10% |
| X/2 | TRBD2*02 8/AAGGTATG/6 TRBJ2-7*01 | n.d. | 0% | 2.43% | 3.17% |
| X/3 | TRBV11-3*01 1/GCCCATAC/2 TRBJ2-2*01 | CASSLGPYNTGELFF | 0% | 2.03% | 1.67% |
| X/6 | TRBD1*01 7/CTTCCCTAGGG/8 TRBJ1-2*01 | n.d. | 0% | 2.55% | 0.70% |
| X/21 | TRBD2*02 5/ACG/4 TRBJ2-7*01 | n.d. | 0% | 7.93% | 0.30% |
| X/23 | TRBV28*01 6/CAGCGAACACGGG/2 TRBJ1-2*01 | CASSSEHGNYGYTF | 0% | 1.21% | 0.28% |
| X/31 | TRBD2*01 2//5 TRBJ2-1*01 | n.d. | 0% | 5.72% | 0.23% |
| X/101 | TRBD2*02 8/ACGA/0 TRBJ2-2*01 | n.d. | 0% | 4.10% | 0.07% |
| B. Cross-reactive clones found in kidney | | | | | |
| III/14 | TRBD1*01 9/G/11 TRBJ1-1*01 | n.d. | 3.59% | 0.23% | 1.32% |
| III/144 | TRBD1*01 2/A/3 TRBJ2-7*01 | n.d. | 1.02% | 0.19% | 0.45% |
| V/43 | TRBV7-9*01 5/CTGACAGGGAGGGTAG/5 TRBJ1-2*01 | CASSLTGRVVGYTF | 0.52% | 1.64% | 1.97% |
| V/71 | TRBD2*02 3/CG/6 TRBJ2-1*01 | n.d. | 0.16% | 0.68% | 1.04% |
| X/99 | TRBD1*01 0//5 TRBJ1-1*01 | n.d. | 0.53% | 0.03% | 0.09% |
| X/102 | TRBV5-6*01 28//36 TRBJ1-5*01 | n.d. | 0.05% | 0.10% | 0.07% |

**References**

1. Haas M, Loupy A, Lefaucheur C, Roufosse C, Glotz D, Seron D, Nankivell BJ, Halloran PF, Colvin RB, Akalin E, Alachkar N, Bagnasco S, Bouatou Y, Becker JU, Cornell LD, van Huyen JPD, Gibson IW, Kraus ES, Mannon RB, Naesens M, Nickeleit V, Nickerson P, Segev DL, Singh HK, Stegall M, Randhawa P, Racusen L, Solez K, Mengel M: The Banff 2017 Kidney Meeting Report: Revised diagnostic criteria for chronic active T cell-mediated rejection, antibody-mediated rejection, and prospects for integrative endpoints for next-generation clinical trials. *Am J Transplant* 18**:** 293-307, 2018
